# Evaluating users’ experiences of electronic prescribing systems in relation to patient safety: a mixed methods study

**DOI:** 10.1186/s12911-020-1080-9

**Published:** 2020-04-03

**Authors:** Lisa Aufegger, Naresh Serou, Shiping Chen, Bryony Dean Franklin

**Affiliations:** 1 NIHR Imperial Patient Safety Translational Research Centre, Imperial College London, London, W2 1PE UK; 2 Centre for Medication Safety and Service Quality, Imperial College Healthcare NHS Trust, London, UK; 3 Tsinghua University, Beijing, China; 4 UCL School of Pharmacy, London, UK

**Keywords:** Electronic prescribing system, User experience, User interface, Eye-tracking, Think-aloud method

## Abstract

**Background:**

User interface (UI) design features such as screen layout, density of information, and use of colour may affect the usability of electronic prescribing (EP) systems, with usability problems previously associated with medication errors. To identify how to improve existing systems, our aim was to explore prescribers’ perspectives of UI features of a commercially available EP system, and how these may affect patient safety.

**Methods:**

Two studies were conducted, each including ten participants prescribing a penicillin for a test patient with a penicillin allergy. In study 1, eye-gaze tracking was used as a means to explore visual attention and behaviour during prescribing, followed by a self-reported EP system usability scale. In study 2, a think-aloud method and semi-structured interview were applied to explore participants’ thoughts and views on prescribing, with a focus on UI design and patient safety.

**Results:**

Study 1 showed high visual attention toward information on allergies and patient information, allergy pop-up alerts, and medication order review and confirmation, with less visual attention on adding medication. The system’s usability was rated ‘below average’. In study 2, participants highlighted EP design features and workflow, including screen layout and information overload as being important for patient safety, benefits of EP systems such as keeping a record of relevant information, and suggestions for improvement in relation to system design (colour, fonts, customization) and patient interaction.

**Conclusions:**

Specific UI design factors were identified that may improve the usability and/or safety of EP systems. It is suggested that eye-gaze tracking and think-aloud methods are used in future experimental research in this area. Limitations include the small sample size; further work should include similar studies on other EP systems.

## Background

Electronic prescribing (EP), also known as computerised provider order entry, has become increasingly common in UK hospitals [1] and in many countries worldwide [2]. One reason for this is its capabilities in incorporating various levels of computerised decision support (CDS). This may include provision of users with on-screen information about specific risks, and/or pop-up alerts to highlight drug-drug, drug-allergy or drug-disease interactions, or duplicate therapy [3]. EP systems can also result in more complete medication orders [4, 5], and have been shown to reduce medication errors [6].

To support patient safety, EP systems are advised to have an appropriate user interface (UI) design, which allows users to interact with the system in a structured, error mitigating and yet intuitive way [7–9]. Aspects of such UI features include, among others, screen layout, density of information, position of messages on the screen, and/or use of colour. Within the clinical setting, previous studies have, in particular, emphasised the importance of alert placement and visibility, screen layout and of information prioritization, including timing and format of decision support alerts [10, 11]. For example, Peikari et al. [12] demonstrated that the self-reported ease of use of the system and information quality (e.g. user interface consistency) can significantly reduce prescribing errors, while Kushniruk et al. [13] highlighted that the risk of medication errors is likely to be increased due to lack of display visibility if prescribers are not able to see the required information on the screen. These studies provide some evidence for a link between medication errors and an EP system’s usability in relation to UI design. However, more research is needed to fully understand the risks and benefits of UI and its impact on patient safety.

Two methods for exploring how users interact with computer screens are eye-gaze tracking and think-aloud methods [14–19]. Eye-gaze tracking is a non-invasive method that gathers data on prescribers’ gaze behaviour and fixation points, and which allows for the interpretation of users’ attentional shifts and cognitive workload (i.e. information processing); think-aloud methods enable researchers to explore the cognitive process involved and to identify areas for improvement. To our knowledge, neither have yet been used to study EP systems.

Our aim was therefore to conduct a preliminary study to explore users’ perspectives of the on-screen design features of a hospital EP system and how these may affect patient safety. Specific objectives were to (1) evaluate the use of eye-gaze tracking to study users’ visual attention and behaviour when interacting with the screen interface; (2) explore prescribers’ experiences of on-screen EP design and how they perceived this to affect patient safety; and, (3) make recommendations for EP system screen design to support patient safety.

## Methods

### Setting

The studies took place at a London teaching hospital organisation using a commercially available EP system, to prescribe for test patients in a simulation setting. The system had some CDS in operation; this included pre-specified order sentences and pop-up alerts for medication prescribed to which the patient had a documented allergy.

### Study design

We conducted a mixed methods study, which took place in two parts. Study 1 was quantitative in nature and applied an eye-gaze tracking method to explore prescribers’ visual attention and behaviour during the prescribing process. Study 2 was qualitative and evaluated the on-screen design of the EP system through a think-aloud technique, followed by a semi-structured interview focusing on the on-screen design of EP systems and their impact on patient safety.

### Recruitment

Participants were recruited via an advert on the organisation’s intranet; any prescriber with experience of the organisation’s EP system was eligible to participate. An information leaflet was given, and consent obtained from each participant prior to the study. No payment was given for participation.

### Procedure

In both studies, participants were asked to complete a prescribing task for a test patient on the EP system, which included prescribing penicillin for an allergic patient. The prescribing task was limited to one medication to keep the process of ordering as standardised as possible.

#### Study 1

In study 1, gaze patterns were recorded during the prescribing process using a Tobii Pro X3–120 integrated screen monitor tracker with a sampling frequency of 250 Hz, equivalent to 250 recorded data samples per second [20]. The screen resolution was 1600 × 1200. This was followed by asking participants to complete a 10-item system usability score (SUS). The SUS is a widely applied, standardised questionnaire, used to understand the degree of a system’s “user-friendliness” [21]. Example items are “I found this system very cumbersome to use” or “I felt very confident using the system”; each item is rated on a 5-point Likert scale (1 = “Strongly disagree” to 5 = “Strongly agree”). Converted into percentiles, a score of 68 (equivalent to the 50th percentile) has been defined as “average” usability [21–23], with a lower percentage suggesting need for UI re-design.

#### Study 2

In study 2, participants were asked to comment on any aspect of the UI whilst prescribing (e.g. how they interpret on-screen design features and what they expect to see and do during the process). This was followed by a semi-structured interview (cf. supplementary material) that explored participants’ views on the UI and the EP system’s usability in relation to medication safety. In particular, we explored (1) how participants viewed the screen and worked through the medical scenario designed for this study; (2) how various design factors influenced their understanding and uptake of information from the screen; (3) what influence these factors may have had on their prescribing behaviour (e.g. navigation, consideration of pop-up alerts, medication selection, etc.); and, (4) what impact (1) and (2) might have on their perception of how the system affects medication safety.

### Data analysis

#### Study 1

Each eye-gaze tracking video was segmented into steps based on the specific tasks and changes in UI features during the prescribing process:

- Step 1: Reviewing patient data (e.g. identity, allergy status) and navigating to the medication page
- Step 2: Reviewing new pop-up window and selecting option to add new medication
- Step 3: Entering drug name in search button and specifying dosages for the medication
- Step 4: Appearance of a pop-up alert and acknowledging it by clicking “OK”
- Step 5: Reviewing the medication order screen and signing
- Step 6: Reviewing final prescription

**Fig. 1:**
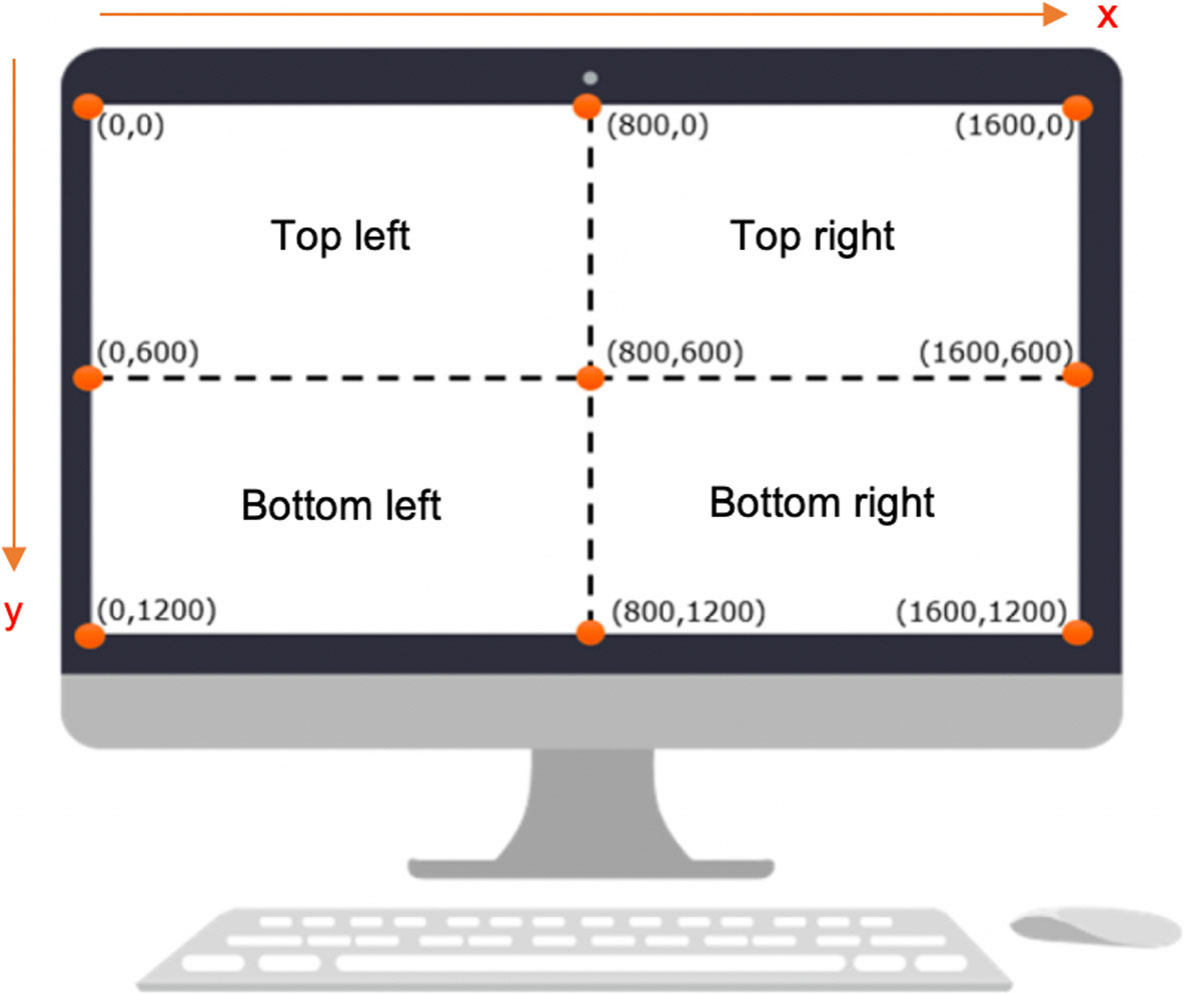
Division of quadrants (created using ProcessOn and Power Point)

The data were exported from Tobii and into the statistical software “R” [24] which was used to process the data for each segment, by exploring gaze behaviour both for the full screen and for each of its quadrants (Fig. 1). By convention, we set the upper-left corner of the screen as the origin (0,0), the top edge of the screen as the X-axis, and the left edge as the Y-axis.

Our main outcome measure extracted was the scan paths (i.e. the mean number of fixation points across all participants) for each screen/quadrant, for each of the segments concerned. Coded as the sequence of items fixated, this measure provides insights into the cognitive state of the user during evaluation tasks, with longer durations indicating an increase in cognitive function (i.e. increased focus, attention, and information processing) [17]. Because of the small sample, we used descriptive analysis to explore both prescribers’ gaze patterns and their perceived usability of the EP system on the SUS.

#### Study 2

For study 2, interviews were audio recorded, transcribed verbatim by an external company, and analysed with NVivo v12 [25], using an inductive thematic approach. Thematic analysis is an exploratory approach and particularly suited for rich yet complex data [26]. Researchers LA and NA familiarised themselves with the data by reading and re-reading the transcripts, before identifying emerging and recurrent themes and key ideas in context relating to the research objectives. The quantitative and qualitative findings from studies 1 and 2 were then synthesised narratively [27] to address the study’s aim.

## Results

### Study 1

The sample for study 1 comprised ten medical prescribers, including five registrars, four foundation year 2 and one foundation year 1 doctors. Participants had, an average, 3.9 (SD = 2.4) years of experience with the EP system concerned. They had the following backgrounds: general surgery (*n* = 1) and medicine (*n* = 1), renal (*n* = 2), orthopaedics (*n* = 1), urology (*n* = 1), plastic surgery (*n* = 1), cardiovascular (*n* = 2), and stroke (*n* = 1). Each eye-gaze tracking session took a mean of 3.28 min (SD = 0.24).

**Fig. 2:**
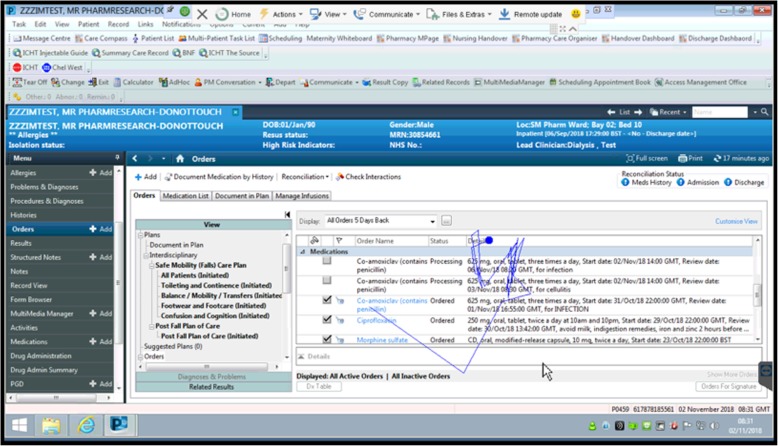
Example of scan path for reviewing medication (created using R version 3.6.1)

During steps 1–3, highest numbers of fixation points were observed in the top and bottom left quadrants (Table 1). These three steps refer to reviewing of patient details, and searching for and ordering the appropriate medication. For step 4, in which the allergy pop-up alert is displayed, most fixations were observed for the top left and the top/bottom right quadrants of the screen, indicating that the prescribers (re-)evaluated the patient data in relation to the pop-up alert. In steps 5 and 6, a large quantity of fixations occurred in the right top and bottom quadrants respectively. In both these steps, prescribers reviewed the medication order before confirming it, with the latter done via an electronic signature request positioned in the bottom quadrant of the right screen. Overall for the full screen, the highest numbers of fixations were observed for steps 3, 4 and 5, indicating that medication selection, review of the medication order, and the pop-up alert led to an increase in number of fixations (Fig. 2) and therefore the highest cognitive load.

**Table 1:**
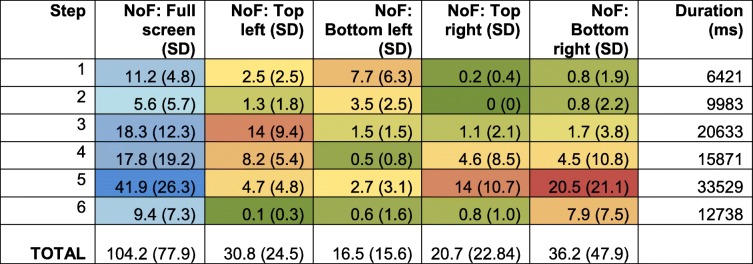
Mean number of fixations (NoF) and standard deviation (SD) across participants for each step: partial and full screen. Green denotes lowest values, yellow to midpoints (50%) between low and high values, and red the highest values. Similarly, for the full screen, low to high NoF are coloured from light to dark blue. The table also provides the video segmented mean duration in milliseconds for each step

In terms of the EP system’s usability; the SUS mean raw score was 39 (SD = 4.7) of 100, with a percentile score of 5% based on cross-industry comparisons, which is considered ‘below average’ in terms of usability. These cross-industry comparisons are based on data from research using the SUS on a wide range of systems and technologies in different contexts and settings [22].

### Study 2

Study 2 comprised ten prescribers, including three registrars, two foundation year 2 doctors, three foundation year 1 doctors, and two pharmacist prescribers. Participants had, on average, 3.3 (SD = 2.3) years of experience in using the EP system. Their areas of expertise were: general surgery (*n* = 1) and medicine (*n* = 1), neurosurgery (*n* = 1), renal (*n* = 2), orthopaedics (*n* = 1), urology (*n* = 1), orthopaedics (*n* = 1) and stroke (*n* = 2). Data analyses from the think-aloud method and semi-structured interviews revealed the following themes: (1) EP design features; (2) usefulness of EP systems; and, (3) suggestions for improvement. Subthemes and example quotes for each theme are presented in Tables 2, 3 and 4.

**Table 2:** Sub-themes and example quotes relating to the theme ‘Electronic Prescribing design features’

Sub-theme	*Example quote*
Pop-up alerts	*“I find, I think they [pop-up alerts], any on screen alert, you’re in danger of getting alert fatigue and you, people tend to close them down and want to move on and not read the detail. So, it’s quite difficult to make them catching.” (Pharmacist)*
Process	*“I think the fact that you have to fill out so many separate fields is challenging, particularly around stop and review dates.” (Registrar)*
Screen layout & design	*“I think it’s too busy just a general feedback, when I’m prescribing. So, you get used to your own places to go out of habit, so I’m ignoring everything else basically.” (Foundation Year 1 doctor)*
Visibility of allergy section	*“I don’t think this [allergy section] is succinct enough in its, I think this should be saying more clearly, this patient is allergic to the drug you’re prescribing, that’s not immediately obvious to me, you have to read a lot to work that out for yourself.” (Pharmacist)*

**Table 3:** Sub-themes and example quotes relating to the theme ‘Usefulness of Electronic Prescribing systems’

Sub-theme	*Example quote*
Accessibility	*“You’ve got access 24/7 wherever you are within the trust [organisation] premises, and you can work remotely, which is an advantage if you, your role entails you* e.g. *going cross site.”(Foundation Year 2 doctor)*
Safety	*“It gives reassurance to the user that what you’re doing is right and the pop alerts make you aware.”(Foundation Year 1 doctor)*
Standardisation	*“It helps, because it standardises the process for a lot of the prescribing that we do. It certainly helps for the drugs that I prescribe that have got complex regimes where they have got additional pre-medication or concomitant medication.”(Pharmacist)*
Keeping a record of relevant information to avoid transcribing	*“Say, for example, the pharmacists have already added the list of medications that patient is already on from the drug history. So, then you just have to click to re-prescribe it, so you don’t have to do it manually every time over and over again. So, I think it is useful to have and it makes your job easier in a way.”(Registrar)*

**Table 4:** Sub-themes and example quotes relating to the theme ‘Suggestions for improvement’

Sub-theme	*Example quote*
Customization for individual prescribers	*“[It would be helpful if] a prescriber can custom their prescribing screen according to the needs like how we do for our Outlook email […] add different folders, basically, arranging and organising information [to] make life easier.”(Foundation Year 2 doctor)*
Incorporation of linking to relevant guidelines	“*I think the trust [organisation] guidelines and policy should be more integrated to this system as I need to minimise this section and go and open a new internet page, search for it and restart again, it is slow, it is a waste of time.”(Pharmacist)*
Pop-up alerts	*“[…] if we do the dose, you get this window, again the option is override, but maybe an option here, if this patient’s allergic to penicillin, would be to view alternatives.”(Pharmacist)*
Use of colour	*“I think the allergy notification at the top left corner could be improved […] it’s not as visual. If it was in a different font or colour, I think it brings the prescriber’s attention […] something you need to think of.”(Registrar)*
Facilitating greater patient interaction	*“For me the biggest disadvantage is that you can easily disengage with the patient very quickly because you could do everything on your desk as a pharmacist. Your ordering, everything can be done just from your desk.”(Pharmacist)*

#### EP design features

Participants referred to design features in relation to pop-up alerts, the process of prescribing, the screen layout and design, and the visibility of the allergy section. The pop-up alerts were discussed in terms of both frequency and specificity. In particular, alerts for mild or redundant interactions were mentioned as a major obstacle and contributor to alert fatigue. The process of prescribing was also discussed in relation to the click-through rate and the lack of automation (e.g. entering fields manually). When it came to navigation and allergy visibility, the screen layout and design were considered too ‘busy’, making the system less intuitive and, thus, a risk to patient safety.

#### Usefulness of the EP system

The usefulness of the EP system was highlighted in association with its accessibility, safety, standardisation, and keeping a record of relevant information which reduced transcribing. For instance, the fact that all information is clearly documented, auditable, and provided in real time was perceived as a facilitator of patient safety.

#### Suggestions for improvement

Lastly, participants expressed a variety of different suggestions for improvement, including customisation, incorporating links to relevant guidelines, and the use of more salient colours. To illustrate, participations wished to have a default list of options of medications based on their speciality, using pop-up alerts as a means to provide alternative solutions to the drug-to-drug interactions, as well as having important information highlighted in colours, bold, or larger fonts. Participants also acknowledged the need to enhance the system’s accessibility to facilitate greater patient interaction; this was due to concerns that prescribing electronically can lead to prescribing remotely, thus precluding patient involvement.

## Discussion

### Key findings

This study aimed to evaluate and create recommendations for UI and on-screen design of hospital EP systems. Findings from study 1 show that, during the overall prescribing process, the highest average number of fixation points was observed during review of patient data, the search, dosage, prescription and order of medication, and the review of the allergy pop-up alert. According to the SUS, the EP system was perceived to be more usable than only 5% of cross-industry solutions. Study 2 revealed three main themes: EP design features, usefulness of EP systems, and participants’ suggestions for improvement. Design features were discussed in relation to their impact on process flow, including aspects such as screen features and layout, as well as information overload. The usefulness of EP systems was expressed in terms of standardization and safety measures that reduce the likelihood of medication errors, while suggestions for improvement were specified in association with embedded prescribing guidelines and changes in system design (e.g. colour, fonts, customization) to enhance information visibility and overall attention.

### Comparison with existing literature

There is wide variation across healthcare organisations and EP system vendors in how on-screen safety information is presented to prescribers, with no clear guidelines derived from studies or best practice recommendations. We know from previous recommendations that placement and visibility and prioritization of information in the design of UI should be customized based on the system [28], and that the colour of alerts or general text information should function as a risk indicator [29, 30], while UI consistency (e.g. buttons that perform the same action and that have the same purpose) is advised to avoid medication errors [10, 12]. Our findings echo these observations, by also highlighting integrated local guidelines, system customisation based on profession (e.g. default medication list commonly used for each speciality) and preference (e.g. layout, font size), as well as enabling greater patient interaction. In addition, and similar to Eghdam et al. [31], findings showed that participants focused on certain screen elements during the prescribing process, reflecting attention on the specific content displayed. However, while Eghdam et al. conducted eye tracking on a prototype that was aimed at supporting antibiotic use in intensive care, we evaluated a fully functioning and operating EP system with particular focus on UI design features, and how these features may affect patient safety.

### Strengths and limitations

A strength of this study is the novel use of eye-gaze tracking to explore a fully functioning EP system, with the focus on patient safety; we have shown this to be a feasible method for studying user interactions with EP. Another strength relates to the qualitative evaluation, which allowed us to explore the limits and benefits of the EP systems, and to design recommendations on how to make the system more efficient. Contributing to the evidence on on-screen design and usability, these findings are an asset for system developers and organisations planning on implementing EP and CDS systems, as well as for those making refinements and adapting existing systems. In contrast, limitations include the small sample size, the use of only one usability measure and the inclusion of only one EP system at one hospital.

### Implication for research and practice

Our findings provide several implications for research and practice. Future studies should focus on collecting data from a larger sample and the application of more usability measures based on aspects such as effectiveness (e.g. success/failure of conducting the task safely) and efficiency (e.g. time on task; click flows). Studies should also evaluate how these affect the usability, perceived satisfaction, and safe use of the system. Studies are furthermore recommended to focus on pop-up alerts and workflow, including aspects such as screen layout and appearance (e.g. colour, fonts, customization of onscreen appearance), and to evaluate their impact on information visibility and overall attention. This will allow further exploration of the degree to which these UI features influence prescribers’ understanding and uptake of information from the screen, leading to evaluation of interventions to improve prescribing behaviour and decision making. We found that use of eye-gaze data was a potentially useful method to evaluate UI features and their impact on patient safety. Eye gaze patterns reflect the prescribers’ visual behaviour during the prescribing task, and, if applied on a large sample, could be used in training, assessment, and feedback. Future studies are therefore advised to explore use of eye-gaze data to compare visual patterns between expert and novice prescribers, and explore its use in improving task performance in relation to safe prescribing. Furthermore, studies are advised to assess the level to which the visual attention during prescribing is a surrogate marker of the desired clinical outcome. Lastly, our findings suggest the importance of working with system vendors to conduct usability studies, in order to evaluate key EP system UI design features with a variety of users, and with the goal of identifying and contributing to evidence-based usability standards for EP systems.

## Conclusion

Creating meaningful user-centred design in EP systems allows participants to interact and work efficiently and safely, and allows for a robust and standardised process of prescribing. System vendors and organisations should recognise the importance of end-user testing and their involvement in all stages of the design, development and implementation of EP systems. The findings in this study provide information about specific UI design features that may relate to the usability of EP systems and improve the accuracy and safety of prescribing.

## Supplementary information

**Additional file 1.** Participant interview.

## Data Availability

The data that support the findings of this study are not publicly available. Because of the nature of the informed consent and ethical restrictions, data distribution is not permitted.

## Abbreviations

UI: User interface
EP: Electronic prescribing
MS: Milliseconds
NoF: Number of Fixations
SD: Standard deviation
Hz: Hertz
