# Supplementary material for: Evaluating users’ experiences of electronic prescribing systems in relation to patient safety: a mixed methods study
Source: BMC Med Inform Decis Mak. 2020 Apr 3;20:62. doi: 10.1186/s12911-020-1080-9 (PMC7126479; doi:10.1186/s12911-020-1080-9)
Supplement: Supplementary file 1 — Additional file 1. Participant interview. [file 12911_2020_1080_MOESM1_ESM.docx]

**Appendix 1- Participant interview**

1. *What is your perception of the on-screen design of the EP systems?*
2. *What is your perception on how the on-screen alerts are displayed?*
3. *What and how do you think information should be displayed in these alerts (drug-drug interactions, layout, etc.)*
4. *When would you display these alerts?*
5. *Where do you think these alert components can be best presented?*
6. *How do you think we can resolve on-screen alerts? If, to what extent to you think on-screen alerts can be improved to facilitate safer prescribing?*
7. *How is the EP system helping and/or hindering your work?*

*Prompt: Have any new problems or issues emerged?*

1. *Can you think of any ways the EP system can be improved?*
2. *Overall, do you think implementation of the EP system has been positive or negative for you as a health professional?*

- *For patients?*
- *For the organization as a whole?*

*Do you have any other comments?*
